# Presenting rose odor during learning, sleep and retrieval helps to improve memory consolidation: a real-life study

**DOI:** 10.1038/s41598-023-28676-z

**Published:** 2023-02-09

**Authors:** Jessica Knötzele, Dieter Riemann, Lukas Frase, Bernd Feige, Ludger Tebartz van Elst, Jürgen Kornmeier

**Affiliations:** 1 Institute for Frontier Areas of Psychology and Mental Health, Freiburg, Germany; 2 Department of Psychiatry and Psychotherapy, Medical Center - University of Freiburg, Freiburg, Germany; 3 Faculty of Medicine, University of Freiburg, Freiburg, Germany

**Keywords:** Psychology, Human behaviour

## Abstract

Improving our learning abilities is important for numerous aspects of our life. Several studies found beneficial effects of presenting cues (odor or sounds) during learning and during sleep for memory performance. A recent study applying a real-life paradigm indicated that additional odor cueing during a Final Test can further increase this cueing effect. The present online study builds on these findings with the following questions: (1) Can we replicate beneficial memory effects of additional odor cueing during tests? (2) How many odor cueing learning sessions and odor cueing nights of sleep maximize the learning success? (3) Can odor cueing also reduce the amount of forgetting over time? 160 Participants learned 40 German Japanese word pairs in four groups with separate experimental conditions over three days. Group N received no odor during the whole study. Group LS received odor cueing during learning and sleep, group LT during learning and testing and group LST during learning, sleep and testing. Participants performed intermediate tests after each learning session plus three final tests 1, 7 and 28 days after the last learning session. Results: (1) Group LST learned 8.5% more vocabulary words than the other groups overall. (2) This odor cueing effect increased across the three days of cued learning. (3) We found no clear evidence for effects of odor cueing on the forgetting dynamics. Our findings support the notion of a beneficial effect of odor cueing. They further suggest to use at least 3 days and nights of odor cueing. Overall, this study indicates that there is an easy, efficient and economical way to enhance memory performance in daily life.

## Introduction

“We are who we are because of what we learn and what we remember”^1^. This sentence nicely summarizes the importance of our brains’ ability to continuously learn and build memories for our self-awareness, and for the perception of and the interaction with our environment (e.g.^2–4^). Memory exists on different timescales, the long-term memory includes memories from years, days and hours ago. The working memory includes memories from a few seconds up to minutes and the ultra-short-term memory (or sensory memory) includes memories from the last second^5^. This classification underlines the importance of memory during every stage of our life^1^. One of the most obvious examples for the role of learning and memory across time scales is the learning and use of first and second languages^6–8^.

Memory formation is typically subdivided into three major steps (e.g.^9^): Encoding (transformation of environmental information into working memory), consolidation (stabilization of working memory content for later retrieval) and retrieval (stored information can be recalled by activating parts of the formed memory trace^10,11^). A large number of studies helped to better understand the basic underlying mechanisms of these steps. Some of the most seminal studies will be summarized in the following:

In 1953, the famous patient Henry Molaison received a bilateral resection of his hippocampus as a treatment of a severe epilepsy. This treatment significantly reduced the number of epileptic seizures. But at the same time it caused an anterograde amnesia affecting his declarative memory (i.e. factual information, previous experiences, and concepts, e.g.^12^). He and similar patient examples demonstrated the importance of the hippocampus for the transfer of short-term memory content to long-term memory^12,13^.

The question of the specific role of the hippocampus for this memory consolidation process was partly answered by animal studies. In 1994, Wilson et al.^14^ recorded a specific pattern of neural activity from electrodes implanted into the hippocampus of rodents, while the animals executed spatial navigation tasks in a maze in order to find food. The authors found a replay of this hippocampal activity pattern during the subsequent slow-wave sleep periods of the animals. Twelve years later, the same group was able to concurrently record neural activity from both hippocampus and neocortex and found neural replay during sleep at both recording sites^15^. According to the authors, the hippocampus is mainly involved in the storage of learned spatial information in working memory. During slow-wave sleep, memory consolidation takes place in a way that the working memory content is reactivated and thereby stabilized to become long-term memory^14,15^. Particularly, the consolidation progress seems to be a function of the extent to which specific memories are reactivated at specific consolidation time windows during sleep^16^. The particular role of the hippocampus during memory formation has been vividly debated since these and other seminal studies (e.g.^17–19^).

During our daily wakefulness we collect a huge amount of information with our senses, but only a tiny part of this information enters our long-term memories. One important question is thus, how our brains determine, what information is relevant and will be stored and what is dispensable and will be forgotten. Emotional labels have been shown to play a role in this process^20^ and also, what repeatedly occurs is worth being remembered^21,22^.

Another interesting and related question is, whether and how we can influence this process from outside. A seminal study from Rasch et al.^23^ indicated that this may indeed be possible to some degree. The study builds upon the finding that the learning context is always associated with the to-be-learned information and becomes integrated in the episodic memory trace^24,25^. Rasch et al. presented rose odor while their participants learned object locations in a two-dimensional memory task. In the subsequent night, the odor was again presented during the participants’ slow-wave sleep (SWS) periods in the first half of the night. In a final test on the next day, the memory was enhanced in this group of participants, compared to a control group, who received a vehicle (placebo) instead of the rose scent. The authors found no effect, when presenting the odor cue during REM instead of during SWS. Likewise, there was no effect when presenting the odor only during sleep but not during learning or alternatively during learning and during the subsequent night while participants stayed awake. fMRI measurements showed that re-exposure to the odor during SWS activated the hippocampus, thus the odor seemed to trigger hippocampal reactivation of the learning content. This reactivation was interpreted to be beneficial for the consolidation process, resulting in better long-term memory^23,26^. The basic idea to associate a secondary cue with learning content and to use this cue to reactivate the related neural activity during consolidation processes during sleep has been termed “targeted memory reactivation” (TMR). TMR effects have been investigated in a considerable number of subsequent studies, including odor and auditory stimuli as cues^16,27–29^.

The present study is based on the results from a recent study by Neumann et al.^30^, indicating that TMR also works in a real-life setting. In their study 11 and 12 years old German school students learned German-English vocabulary in their school environment and at home. In four experimental conditions participants received rose odor cues (1) during learning and during sleep (LS), (2) during learning and during a final test (LT), (3) during learning, sleep and a final test LST) and (4) no odor at all (control). Neumann et al. replicated the findings from Rasch et al. of better memory performance with odor cues during learning and during subsequent sleep (Condition 1) compared to no odor (Condition 4). Remarkably, Condition 3 with odor during learning, sleep and during the Final Test – an experimental condition that had been realized for the first time in this study – provided the best memory performance results with the largest effect size (LST group vs. control group: d = 1.22; LS group vs. control group: d 0.64). Thus, odor cueing seems to improve not only memory consolidation but also memory retrieval.

The TMR effect offers an efficient way to optimize learning and Neumann et al.’s study provides strong evidence that it can easily be applied in real life. A recent real-life study by Göldi and Rasch^31^ applied auditory cues in a TMR design. The authors reported heterogenous results, partly due to sleep disturbances from the auditory stimulation. In the present study we keep our focus on olfactory cues and address some questions we derived from Neumann et al.:

1. Can beneficial memory effects of additional odor cueing during tests be replicated?
  Neumann et al. showed for the first time that odor cueing during all of the phases (learning, sleep and testing) provides maximal memory performance. In the present study we will investigate whether this can be replicated.
2. How many odor cueing learning sessions and odor cueing nights of sleep maximize the learning success?
  Participants in the Rasch et al. study had one TMR learning period and one TMR night. In Neumann et al. participants had 7 days and nights between the first introduction of the vocabulary learning material and the final test. However, the authors did not control for the actual number of learning sessions across these 7 days in this real-life setting. Participants in the current study had three learning sessions on three subsequent days with three nights of sleep and an intermediate test at each day. This allowed us to investigate whether the TMR effect can be even increased across days.
3. Can odor cueing also reduce the amount of forgetting over time?
  In the current study we were also interested in whether odor cueing not only increases the amount of memorized learning content but also the stability of the memory engrams. A recent study by Rakowska et al.^32^ applied auditory cues in the context of a motor sequence learning task and applied final tests one day, ten days and six weeks after the last learning block. They found a significant TMR benefit after ten days but not after one day or six weeks. To our best knowledge no TMR-study using declarative learning material tested for longer-term memory effects. In order to test for long-term memory effects with declarative learning materials, we compared three final tests, one after 24 h, another after one week and a third final test after one month.

## Methods

### Participants

183 healthy participants completed the whole experiment.

The participants declared that they did not have any symptoms suggestive of amnesia, a limited olfactory function or severe sleeping problems. In addition, they declared that they were not taking any sedative drugs nor consumed alcohol during the experiment and 24 h in advance. The participants were between 18 and 35 years old and were native German speakers. In addition, they were not allowed to have any prior knowledge of the Japanese language.

The study was performed in accordance with the ethical standards laid down in the Declaration of Helsinki^33^. Moreover, an ethical approval was given by the local ethics committee (Ethik-Kommission der Albert-Ludwigs-Universität Freiburg). Participants gave written informed consent to participate in our experiment.

### Online program

The Gorilla Experiment Builder was used to perform this online study. Gorilla is a cloud-based research platform with different integrated builders to create behavioral experiments (https://gorilla.sc/). The program can be accessed via a web browser (Anwyl-Irvine et al.^34^).

### Questionnaires–exclusion criteria

Before the actual experiment started, the participants filled out two separate questionnaires. The first questionnaire served to verify that none of the exclusion criteria was met. As a second questionnaire, the Pittsburgh Sleep Quality Index (PSQI), was used to exclude participants with severe sleeping problems. 195 participants with a score of less than ten were included. 183 participants completed the whole experiment, 18 participants had to be excluded as they didn’t follow the instructions (they forgot to place the odor next to them or didn’t stick to the given time intervals). Thus, 165 participants could be included in the analysis.

### Vocabulary

For this study, 40 different Japanese-German word pairs were used (the wordlists can be seen in Table S1 in the Supplementary Materials) 20 of these words were nouns, 10 adjectives, and 10 verbs. This selection was based on the one used by Kornmeier et al. in 2014^21^. All words (German and Japanese) were at least disyllable. We used common Japanese words with a clear German translation. All 40 Japanese-German word pairs were presented in each learning session and were retrieved in each testing session.

### Experimental paradigm

**Figure 1:**
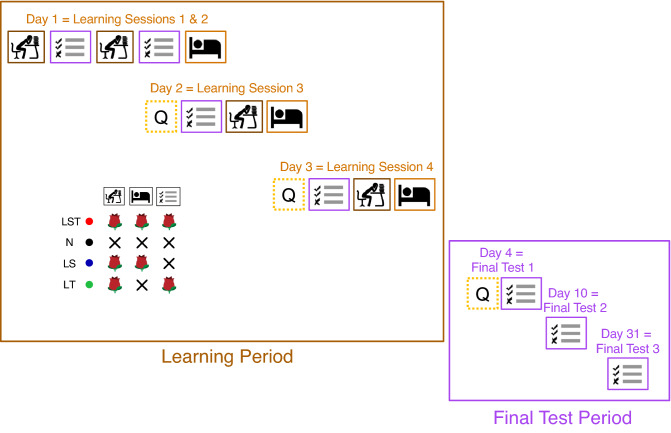
Experimental setup. Day 1 consisted of 2 Leaning Sessions, with one Learning Session consisting of a learning part which was followed by a testing part. Day 2, 3 and 4 started with the SFA questionnaire (Q). On day 2 and 3, the questionnaire was followed by the Learning Session, which started with the testing part and was followed by the learning part (order is switched compared to day 1). On day 4 the Final Test period started after the questionnaire with the first Final Test. Participants conducted Final Tests on day 4, day 10 and on day 31. The table with pictograms on the bottom left summarizes the four experimental conditions. The icons therein are adopted from Neumann et al. (2020).

The main goal of this study was to further examine the effects of odor cueing on memory consolidation. To serve this objective, four different conditions were tested. The conditions differed in the application of rose odor during learning, testing and sleep (see table in Fig. 1). The N Condition (**N**o odor) was the control condition in which no odor was applied in any phase. The median age of participants in this condition was 24 with a range between 18 and 33 years, 9 participants were males. In the LS (odor during **L**earning and **S**leep; participants’ median age was 25 with a range between 19 and 34 years, 8 male) and LT Condition (odor during **L**earning and **T**esting; participants’ median age was 25 with a range between 19 and 35 years, 10 male), participants received odor during learning and either during sleep (LS) or during testing (LT). Participants of the LST Condition (**L**earning, **S**leep and **T**esting; participants’ median age was 23 with a range between 18 and 31 years, 8 male) received rose odor during learning, testing, and sleep. Importantly, in Conditions LT and LST the odor cues were presented during Intermediate Tests–within the Learning Period–as well as during the final tests in the Final Test Period.

The participants were randomly assigned to the different conditions. The participants were not informed about the condition in which they were tested.

### Odor cue

For this study, odor sachets containing rose odor from the American company Greenleaf (Greenleaf Scented Envelope Sachet Roses) were used. The sachets contained purified, freeze-dried mineral clay, mixed with the fragrance “Quiddity” from Greenleaf. The sachets can be bought conventionally in drugstores or via the internet and are harmless to humans’ health. The aroma lasts up to three months. We transferred the material out of the small sachets, into teabags. The teabags were put into small, grey envelopes (6 cm × 9 cm) with the labels “Lernen” (German translation for learning) in yellow, “Testen” (testing) in red, or “Schlafen” (sleeping) in blue. This transfer was done to make sure that all participants, regardless of the condition, had three similar envelopes with three labels (“Lernen”, “Testen”, “Schlafen”). The sachets without odor contained unscented scrap of paper instead of odor material in order to provide similar appearance and weight of sachets with and without odor. Participants of the LST Condition received for example three sachets with odor, participants of the LS Condition received the sachets “Lernen” and “Schlafen” with odor and the sachet “Testen” with scrap of paper. We sent the sachets to all our participants and instructed them to place the sachets next to themselves during the respective time points.

### Task and experimental procedure

The actual experiment consisted of six blocks performed on separate days (see Fig. 1). On the first day, two Learning Sessions were completed to make sure that enough vocabulary was learned and to prevent potential floor effects. One Learning Session consisted of a learning part (about four minutes) and a testing part (about five minutes). During the learning part, the vocabulary pairs were presented each for three seconds on the screen in a random order. During the testing part, the Japanese words were presented in a random order on the screen and the participants had to recall and type in the German translation within nine seconds. After each inquiry, the correct German translation was presented on the screen for two seconds as feedback. This feedback was presented during each testing part (during the Learning Period as well as during the Final Test Period).

Day 2 to day 4 started with a questionnaire (Q) about the sleep quality of the night before (SFA questionnaire = Schlaf-Fragebogen A^35^). After completion of the questionnaire, on days 2 and 3, a Learning Session with reversed order followed: Participants had to do the testing part first, to measure how many vocabularies they were able to recall from the Learning Session the day before.

The Final Tests took place on day 4 (one day after the last Learning Session), day 10 (one week after the last Learning Session) and day 31 (four weeks after the last Learning Session). The Final Test period contained only testing parts and no learning parts. The participants were instructed to perform each block in the morning. The minimum time interval between the different blocks had to be 24 h to keep the temporal context as stable as possible across days. It was not possible for the participants to shorten these intervals, e.g. it was impossible to start day 1 at 8 AM and day 2 at 6 AM.

Participants were instructed to place the odor envelopes right next to them:

- The “Lernen” sachet during the Learning parts of days 1, 2 and 3
- The “Schlafen” sachet during all nights between day 1 and day 4
- The “Testen” sachet during all Intermediate Tests and the Final Tests

Participants were further instructed to ventilate and change the room and to place the odor sachets in airtight zip lock bags (“Noak-bags”, see acknowledgement) after the respective part, to make sure that they don’t smell the rose odor between the different parts.

### Data analysis

The data analysis was executed in Igor Pro Version 8.0.4.2 (Wavemetrics Inc., Lake Oswego, USA) on a Windows 10 Pro computer.

### Comparisons

As a measure of memory performance, the amount of correct vocabulary entries was assessed. For the identification of a correct entry different criteria were applied. According to the strictest criteria the recalled words had to be typed correctly, only the usage of small instead of capital initial letters or “ss” instead of “ß” were not taken into account. For a liberal criterion, a threshold for the minimum needed number of matching letters between the entry and the correct answer was defined in order to regard the word as learned. For this criterion the order of these letters had to be also correct. In the most liberal criterion, an additional threshold was set to define a maximum number of allowed scrambled letters between the entry and the correct answer in order to regard the word as learned. We did not find significant differences between these measures and thus took the strictest criterion for our analysis.

For the statistical analysis of the intermediate tests during the learning period (including Final Test 1 on day 4) we fitted the data with linear equations. We took Test 1 (T1) as a starting point for this analysis, because T1 was followed immediately after all 40 vocabulary pairs had been presented only once and T1 was before the first night of odor cued sleep (for LS and LST). Thus, all four groups should show about equal performance. For this linear fit analysis, we did not take the results from the immediately following T2 into account, as it was the only test that had to be performed in the immediate succession to the previous test (T1) on the same day and particularly before the first night of consolidative sleep. Further, T2 was the only test that followed after the vocabulary material had been observed two times in immediate succession. We will discuss potentially critical aspects of this choice of a starting point in a separate paragraph in the Discussion section.

The Holm-Bonferroni method^36^ was applied to correct for multiple testing, if not otherwise specified. Corrected and uncorrected p-values are reported in the following. We further used non-parametric permutation tests^37^ to compare performance between conditions. The significance threshold was set at α = 0.05.

The basic idea of a permutation test is to generate reference distributions out of the measured data instead of relying on theoretical distributions. This is done by permuting the assignment of the measured data to the experimental groups. If the total number of theoretically possible permutations is too large concerning calculation time, only a random sample of permutations can be selected. In this case, the test is called “randomization test”. We applied randomization test variants and used 1000 permutations.

The results of the Final Test Period were analyzed with two mixed model ANOVAs.

### Data pre-processing

This study consists of a between-design with four experimental conditions. In order to minimize the potential influence of a priori differences between experimental groups on the study outcome, an outlier detection was done before the relevant experimental interventions took place.

This was done in the following way:

1. All data from the second test of the first learning day (T2) (i.e., before the first night) were pooled together and the mean and standard error of the mean (SEM) were calculated
2. Two thresholds were defined, excluding the bottom and top 5%
3. These thresholds were applied to each of the four data sets (from the four conditions) and all participants above the top threshold and below the bottom threshold were excluded

**Figure 2:**
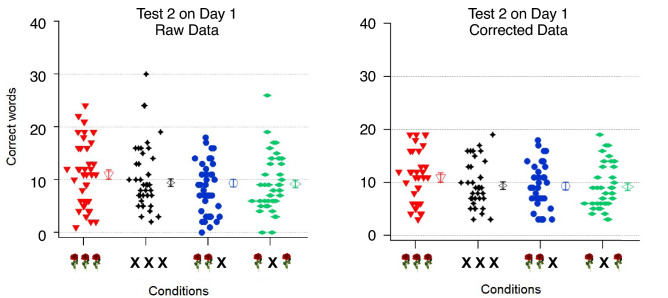
Results from Test 2. The left graph shows the data of all participants (small, filled icons). The right graph shows the outlier-corrected data, as described in the main text above. The different experimental conditions are shown on the x-axis (from left to right: LST in red, N in black, LS in blue and LT in green). The y-axis indicates the number of correctly recalled words. The open icons on the right of the icon clouds represent the grand means ± SEMs of the respective conditions. N: no odor stimulation; LS: odor stimulation during learning and sleep; LT: odor stimulation during learning and during the tests; LST: odor stimulation during learning, sleep and the tests.

Figure 2 displays the original (left) and corrected (right) data distributions from the different experimental conditions. Table S2 in the Supplementary Materials presents means and SEMs before and after correction. T2 was selected for this preprocessing step, as during T1 the differences in performance were not yet as clear (most of the participants had less than five correct words). During T2, some participants already had odor right next to them (Condition LST, LS, LT), but following the results of Rasch et al.^23^ and Neumann et al.^30^, odor during sleep is necessary in order to gain a beneficial effect in memory performance. Thus, T2 was selected to identify and reduce the a priori unspecific differences between the experimental groups dedicated to the four different conditions. Eight participants of Condition LST were eliminated, 4 participants of Condition N, 5 participants of Condition LS and 2 participants of Condition LT.

The scatter plots in Fig. 2 together with the statistical data (see Table S2 in the Supplementary Materials) indicate that the experimental groups are more homogenously distributed after the correction (Fig. 2, right; see also Fig. S1 for a more detailed comparison between the raw and manipulated data). Hence, all following results are based on this corrected data (Fig. 2, right).

## Results

**Figure 3:**
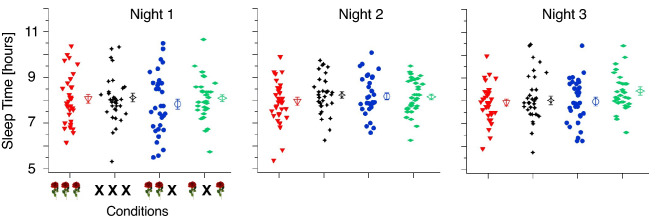
Sleep times. The sleep times for all participants (of the corrected data) of each condition are shown separately for the first three nights. The different conditions are displayed on the x-axis, LST in red, N in black, LS in blue and LT in green. The sleep time in hours, as reported by the participants, is shown on the y-axis. Each filled icon represents data from one participant. The open icons on the right-hand side of each cloud of illed icons represent the grand means (larger unfilled icons) ± SEMs.

We first checked for possible confounding factors: There was no significant difference in median age or gender between conditions. Moreover, no significant differences between conditions regarding sleep times of the first three nights (see Fig. 3 and Table S3 in the Supplementary Materials) nor regarding the starting time of the Learning Sessions across days (see Fig. S1 and Table S4 in the Supplementary Materials) were found.

### Memory performance and linear fits

Different types of mathematical models for learning dynamics are discussed in the literature, the most common type is the psychometric (sigmoid) function^38^. The present learning data most likely reflect the linear part of the psychometric function, therefore a linear and a sigmoid fit function were applied to fit the data. The related goodness of fit (R square) was calculated for the linear and the sigmoid fit functions separately for each individual participant. Given that linear and sigmoid fits performed equally well (both above 98% explained variance on average), the linear fit was chosen for simplicity reasons.

In detail, the data sets were separated into a learning period (Intermediate Tests T1, T2, T3, T4 and Final Test F1) and a final test period (F1, F2 and F3). In order to compare the learning dynamics between experimental conditions, linear fits were applied to the number of correctly reproduced vocabulary across the four time points T1, T3, T4, and F1, separately for each individual participant. The line slope was used as a dependent variable for statistical tests between experimental conditions.

The slopes of all line fits are positive (see Table 1), indicating a rising learning progress across conditions. Importantly, the line slope from Condition LST is significantly larger than the slopes from the other three conditions (N vs. LST: *p* = 0.037, LT vs. LST: *p* = 0.0029; LS vs. LST: *p* = 0.028; see Table 2), indicating a steeper rise of the memory performance across days. No significant differences between the line slopes of conditions LT, LS and N were indicated. The results of the uncorrected and corrected p-values (Holm-Bonferroni method) are displayed in Table 2.

**Table 1:** Basic statistics of slope values. The basic statistic is shown for each condition separately (from left to right: LST, N, LT, LS). N_p_ represents the number of participants included in the condition. The Mean, SD (standard deviation), SEM (standard error of the mean), Max (maximum slope), Min (minimum slope) and the Median are listed. LQ shows the lower quartile and UQ shows the upper quartile.

LST	N	LS	LT
N_p_: 34	N_p_: 37	N_p_: 36	N_p_: 39
Mean: 6.9824	Mean: 5.9541	Mean: 5.8917	Mean: 5.4256
SD: 2.1059	SD: 2.3014	SD: 2.619	SD: 2.3267
SEM: 0.36116	SEM: 0.37835	SEM: 0.4365	SEM: 0.37257
Max: 11.2	Max: 11.2	Max: 11	Max: 10
Min: 3.3	Min: 2.6	Min: 1.3	Min: 0.9
Median: 6.7	Median: 5.9	Median: 5.4	Median: 5.5
LQ: 5.35	LQ: 4.2	LQ: 4.025	LQ: 3.95
UQ: 8.275	UQ: 8	UQ: 7.475	UQ: 6.8

**Table 2:**
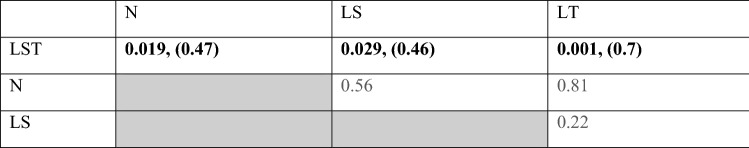
Permutation test statistics of the slope parameter between conditions. Listed are *p* values and Cohen’s d for significant *p* values in parentheses. Significant *p* values (alpha = 0.05, with Bonferroni-Holm correction) and effect sizes in bold.

**Figure 4:**
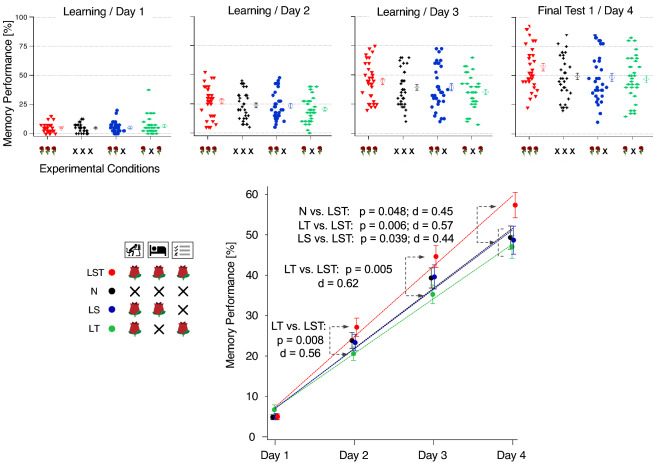
Results of the Intermediate Tests during the learning period. Top: Scatter plots of test results across four days. Filled icons represent data from individual participants, larger open icons are grand means within experimental groups (condition) ± SEMs. Bottom: Grand means per condition and day with linear fits and results from the post-hoc tests. Please notice that the presented fits are only for demonstration purposes. For the data analysis we used the slope parameter from individual linear fits separately for the individual participants. d: Cohen’s d.

The scatter plots of all tests within the learning period for each condition are shown in Fig. 4, upper part. The lower part of Fig. 4 displays the grand means ± SEMs of the different experimental conditions together with linear fits.

The first and second Intermediate Tests on Day 1 were executed directly after the respective learning blocks and thus without a night of sleep with possible odor cueing. All other Intermediate Tests were executed after a learning block and a night of sleep. If odor had to be applied during sleep, it was presented during sleep after Day 1, Day 2 and Day 3 (see the experimental design in Fig. 1). On Day 4, participants of the LST Condition gave the correct German translation for 57% of the Japanese words on average. In conditions N and LS, correct responses were given for 49% and in condition LT for 48% of the words on average. Overall, participants of the LST condition memorized 8.5% more vocabulary words on average compared to participants of the other conditions. Moreover, participants of the N and LS conditions seem to be similar in performance, whereas participants of the LT Condition seemed to perform worst (Fig. 4), although the slopes of these three conditions did not significantly differ.

To investigate at which test the LST Condition starts to differ significantly from the other conditions, post-hoc permutation tests were calculated separately for each time point (see Table 3 and Fig. 4, lower part). These post-hoc tests between experimental conditions are statistically dependent on the slope statistics, presented above (Table 2) and are thus listed without multiple testing correction (for comparison: post-hoc permutation tests between conditions for the uncorrected data can be found in the Supplementary Materials in Table S5).

**Table 3:**
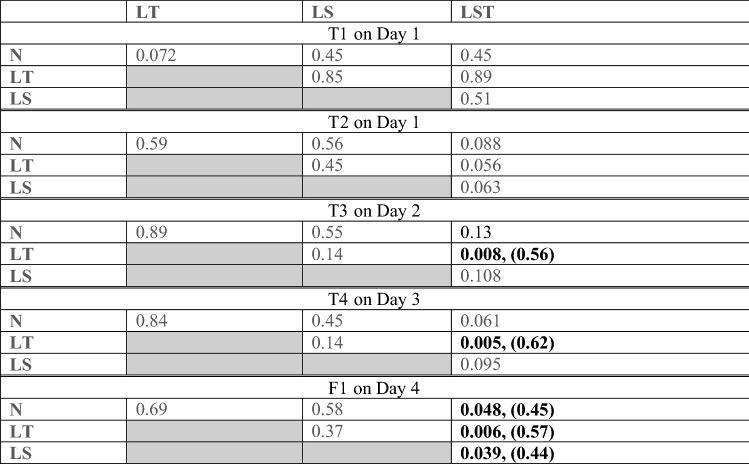
Uncorrected post-hoc tests for Intermediate Tests T1, T2, T3, T4 and Final Test F1. The results of the permutation tests are displayed for each test separately (from top to bottom: T1, T2, T3, T4, F1). Non-significant *p* values are presented in grey, the statistically significant *p* values and effect sizes (Cohen’s D in parentheses) are displayed in bold black.

Already at Day 2 (after the first sleep Session with odor), participants of the LT Condition and participants of the LST Condition differ significantly (*p* = 0.008) in performance. The same is true for Day 3 (after two nights with odor; *p* = 0.005). At Day 4, the performance of participants in the LST Condition is significantly better than the performance of participants of all the other conditions (N vs. LST: *p* = 0.048, LT vs. LST: *p* = 0.006; LS vs. LST: *p *= 0.039). No significant differences in none of the comparisons can be found between LT, LS and N, confirming the slope statistics as reported above.

### Forgetting dynamics

The Final Test 1 was conducted one day after the last learning block (after the last night with odor for Conditions LST and LS), Final Test 2 one week, and Final Test 3 one month after the last learning block. We took Final Test 1 as the starting point to analyze the forgetting dynamics.

**Figure 5:**
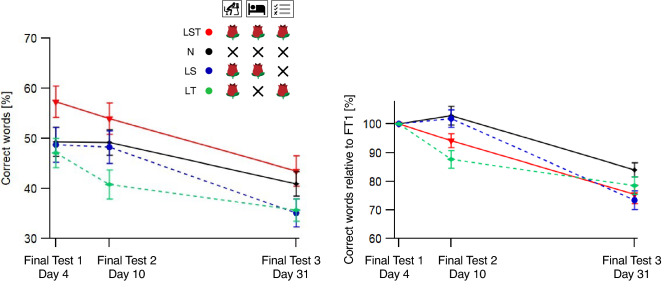
Left: Mean percentage of correct words ± SEMs for all conditions and Final Tests. Right: Mean percentage of correct words relative to Final Test 1 ± SEMs for all conditions and Final Tests. On the x-axis, the different tests are displayed (Final Tests F1 on Day 4, F2 on Day 10, F3 on Day 31). The LST Condition is displayed in red, the N Condition in black, the LS Condition in dashed blue and LT in dashed green.

Figure 5 presents the results (grand means ± SEM) of the three Final Tests in two different ways. The left graph shows the average percentage (grand mean across participants) of memorized word pairs per Final Test, separately for the four experimental conditions (different colors). The right graph shows the same data normalized with respect to Final Test 1 (for each participant, the results from Final Tests 2 and 3 were divided by the results from Final Test 1).

In contrast to the learning dynamics as presented above, the forgetting dynamics did not provide consistent patterns across participants to apply a common mathematical fit function. We thus decided against using data fitting parameters for the statistics and instead analyzed the raw Final Test results (with different starting values for the forgetting analysis) and the normalized Final Test results (results from Final Tests 2 and 3 divided by the results from Final Test 1, making starting values for the forgetting analysis equal) with separate ANOVAs with the factors TIME, with three (two for the normalized data) steps according to the three (two) Final Tests, and CONDITION, with four steps according to the four experimental conditions. Both ANOVAs revealed a strong effect for the factor TIME (Raw Data: F(2284) = 145.3, *p* < 0.0001; Normalized Data: F(1142) = 151.4, *p* < 0.0001) reflecting – non-surprisingly – forgetting dynamics over the four weeks. For the raw data, the factor CONDITION was not indicated as significant (F(3142) = 2.5, *p* = 0.061), for the normalized data, it reached significance (F(3, 142) = 3.18, p = 0.026). Both ANOVAs indicated significant interactions (Raw Data: F(6142) = 4.2, *p* < 0.0001; Normalized Data: F(3142) = 7.2, *p* < 0.0001). Based on these results we calculated separate post-hoc permutation tests for each Final Test (Raw Data: FT1, FT2, FT3; Normalized Data: FT2, FT2) with Condition as the dependent variable. We found significant effects only for FT2 (Raw Data: FT1: *p* = 0.09; FT2: *p* = 0.02; FT3: *p* = 0.07; Normalized Data: FT2: *p* = 0.0005; FT3: *p* = 0.08). We calculated additional post-hoc permutation tests to unpack the effects from FT2. The results are listed in Table 4.

**Table 4:**
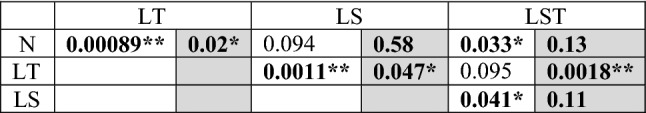
Post-hoc permutation tests of the data from Final Test 2. Data were tested in two versions: raw data and normalized data (grey fields). Stars indicate significance after Bonferroni-Holm correction for the number of orthogonal tests with *p* < 0.05* and *p* < 0.01**.

## Discussion

The present online study focuses on the targeted memory reactivation (TMR) with rose odor cues building on a recent field study by Neumann et al.^30^. We found superior memory performance in the experimental condition with odor cues during learning, sleep and during the tests compared to the other three experimental conditions. Further, the identified TMR effect increased across three Learning Sessions and nights of sleep with odor cueing. Several studies reported also TMR effects in a condition with odor cueing during learning and during sleep (but not during retrieval), compared to a number of control conditions e.g.^23,27,30^. We did not replicate this finding. We additionally analyzed whether odor cueing also affects the forgetting dynamic across four weeks but found no clear effects.

### Limitations of this study

#### Limited control of experimental parameters in an online study

The present study was executed as an online study. Participants received the envelopes containing either the odor materials or the scrap of paper (control conditions) by mail and were instructed to put them on the desk while they learned and were tested, and on their nightstands while they slept. We were not able to control how seriously our instructions were realized at the participants’ home, and how the exact distance was between the odor cues and the participants’ noses during learning, testing and sleep. We were also unable to control how good the participants followed our instructions to ventilate the room after the respective parts and to execute the different parts in separate rooms, if necessary.

Participants filled out questionnaires about the quality and duration of their sleep during the learning period. This controls the important factor sleep to some degree but not as precise as if it were monitored in a sleeping lab.

#### Limited control of participants cooperation in an online study

In a typical experiment in the lab the participants and experimenters are in direct contact. Cases of poor cooperation of participants are rare and can be relatively easy detected in the lab. It is much more difficult to control the cooperativity of participants in an online study, even more if the study is extended over several days. In the present study we preprocessed our raw data to also address this problem (see “Methods” section). However, there may still be a variability of the participants’ cooperativity between conditions. This may affect the quality of the measured data and potentially reduce effect sizes.

#### Odor presentation

The presentation of odor cues during the whole night (instead of presentation only during specific sleep stages) has the advantage that TMR can also easily be used at home as no identification of sleep stages in a sleep laboratory is necessary. However, this introduces some confounding factors. The odors are not only presented during sleep, but also during some time when participants are awake, just before and just after sleep. Moreover, the total time during which the odors are presented is much longer in the LST and LS conditions compared to the LT and N conditions. It is not clear if those factors may also have an impact on the results.

#### Potential confounding factors for Intermediate Test 1 and 2 results

The first day contained two Learning Sessions in immediate succession, which is in contrast to the subsequent days within the Learning Period with only one Learning Session per day. We did this in order to prevent potential floor effects (see Task and Experimental Paradigm in the Methods section). It turned out that the learning performance from T1 to the immediately following T2 (no sleep and no odor between T1 and T2) was much larger than from T2 to T3 (with sleep and odor treatment in between in conditions LS and AL), where the odor cueing effect should be visible. We currently explain this observation with the so-called “Testing-Effect”. The Testing Effect is a well-known memory effect which indicates that successive tests can particularly improve learning performance^e.g.^^21,39^.

Further below we will speculate about a limited learning capacity within a certain time range and that this limit may have already been reached by the Testing Effect at the end of Test 2 for the time range between Test 1 and Test 3. As a consequence, the impact of the odor treatment in the first night may be very limited. This may explain that larger amount of learning performance took place between Test 1 and Test 2 on Day 1, but not between Test 2 on Day 1 and Test 3 on Day 2 (see also Fig. S2 in the Supplementary Materials).

The Testing Effect is thus a potential confounding factor for the present data. Taking this into account, we have three different options for the linear fits of the learning performance across days:

- Taking the results from Test 1 of Day 1 as a common starting point for the linear fits of the four experimental groups. At this point all four experimental groups had seen the vocabulary material only once and showed no difference in memory performance, indicating that they had comparable vocabulary knowledge states. We report the results of this strategy in the Results section above. A consequence of using Test 1 results as starting point is of course, that the performance gain from Test 1 to Test 3 in Fig. 4 cannot be directly compared with the performance gains across the subsequent tests. However, the results from Test 1 serve as a common starting point across groups before a certain amount of additional learning. The major finding in the present study, i.e. the differences between the Condition LST and the other Conditions can hardly be explained by this initial confound, particularly because the largest difference between Condition LST and the other conditions can be observed at the Final Test 1 on Day 4.

b. Taking the results from Test 2 of Day 1 as a common starting point for the linear fits of the four experimental groups. At the end of Test 2 participants are in the last vocabulary-knowledge state before sleep with odor cueing in conditions (LS and AL). Results from this analysis variant can be found in Table S6 in the supplementary materials.
c. Fitting only the results from (intermediate) Test 3 on Day 2 to Final Test 1 on Day 4. This circumvents the difficult situation of Day 1. However, potential influences from the Testing Effect remain, simply because the Testing Effect can be present in all results from Test 2 to Final Test 3. We discuss this point further below in more detail. Results from this analysis variant can also be found in Fig. S3 and Table S6 in the supplementary materials.

None of the three approaches solves the problem completely. A follow-up study needs to add experimental groups keeping the odor treatment over days but realizing only one intermediate test per group.

Interestingly, neither for Test 1 nor for Test 2 we found a significant difference between the four experimental conditions. However, for Test 3 on Day 2, i.e. after sleep and differential odor treatment, we do find a first significant difference between the conditions LT and LST, indicating already an impact of the odor treatment in the night between Day 1 and Day 2 beyond the above mentioned confound (see Table 3 and Fig. S1 in the Supplementary Materials). Of course, this finding needs to be taken with caution because the statistical results in Table 3 are uncorrected, but it fits into the major finding of this study.

### Can beneficial memory effects of additional odor cueing during a final test be replicated?

Several studies about TMR focused on cueing during different sleep periods at night (e.g. SWS, REM^23,40,41^, on different cueing modalities (olfaction^23,30^, audition^31,40–42^), and learning material (memory game^23,40^, vocabulary^30,42^, motor learning^23,41^, etc.). Odor cueing seems to work best with declarative learning materials (e.g. vocabulary^30,42^) and with cueing either during slow wave sleep (SWS^23^) or during the whole night^30^. While the majority of studies focused on cueing during learning and during sleep, Neumann et al.^30^ were the first to ask whether odor cueing during memory retrieval is also beneficial. They investigated this question with two additional conditions. In one condition odor cueing was realized during learning, sleep and during a Final Test (Condition LST). In a second condition odor cues were only presented during learning and during the Final Test but not during sleep at night (Condition LT). Neumann et al. found evidence for additional performance benefits in the LST Condition, compared to a condition with odor during learning and sleep (Condition LS). Moreover, the conditions LS and LST were superior to the LT Condition and to a condition with no odor at all.

One aim of the present study was, to test, whether the beneficial TMR effect of additional odor cueing during the Final Test in the LST Condition can be replicated. In our study the LST Condition produced better memory performance than the three other experimental conditions. Particularly, we replicated our previous findings of better memory performance in the LST Condition than in the LT Condition. This indicates that cueing during learning (encoding) and sleep (consolidation) are necessary preconditions for the beneficial effect of cueing during retrieval (during the tests).

### How many Sessions (learning periods and nights of sleep) with odor cueing are necessary to maximize the TMR effect?

The majority of TMR studies contained one Learning part, one night of sleep and one Final Test the next day. Neumann et al.’s field study was executed with school children in their schools and at home. The study was realized as part of the regular school curriculum with one experimental condition per English-German textbook unit. Consequently, the new vocabulary unit was introduced at day one and the Final Test took place after one week. Thus, the students had theoretically seven days of learning and seven nights of sleep between the first Learning Session and the Final Test. How many Learning parts they practically executed, was not controlled. Neumann et al. replicated previous TMR findings with comparable effect sizes (plus additional findings concerning the LST Condition, as described above). However, up to date it is an open question, whether the TMR effect can be increased with more than one learning (and sleep) Session with cueing and how many of such Sessions are optimal.

In the current study we introduced three learning (and sleep) parts on three successive days (and nights). We further executed intermediate tests after each night in order to measure the learning dynamics across the three days. Our results indicate that the benefit of odor cueing increases over these three days, as indicated by the larger  slope value for the LST condition compared to the other conditions.

Göldi and Rasch^31^ also had three days of cued learning combined with three nights with cue stimulation. They also observed the largest effects after three days, although they used auditory cues and their overall results are heterogenous, most probably because the sleep of some participants was disturbed by the auditory cues.

So far it is unclear after how many Learning Sessions (a session consists of a test and a learning part) the memory benefit reaches its ceiling. This depends most probably also on the amount of learning materials with fewer Learning Sessions in the case of fewer vocabulary material and vice versa. The learning material as such, whether it consists of vocabulary pairs, a memory game (as e.g., in Rasch et al.^23^) or e.g., mathematical content, may also play a role in this context. These relevant questions need to be addressed in follow-up studies.

### Can odor cueing also reduce the amount of forgetting over time?

Before our study was conducted, all the studies on the TMR effect measured learning success at one Final Test after the learning period. This indicates how much of the learning content has been transferred into long-term memory stores. However, results from one Final Test do not tell us how stable the long-term memory engrams are across time. We tested this by introducing three Final Tests, 24 h, one week and one month after the last Learning Session.

As expected, we found forgetting over time across all experimental conditions. The amount of forgetting after four weeks was between 15 and 25% with respect to the first Final Test after 24 h. Beyond the expected overall forgetting across conditions, the indicated significant interaction between the experimental conditions and the Final Test Days and the results from the subsequent post-hoc tests can be drawn back to differences at Final Test 2, i.e. one week after the last Learning Session. Both, the analysis of the raw data and the analysis of the normalized data revealed the worst performance for the LT condition. The indication of superiority of the LST condition in the raw data analysis can easily be traced back to the higher level of learning performance of the LST condition, as indicated by the analysis of the Learning Period. Normalizing for this effect, in contrast, indicates less forgetting for the conditions N and LS, than for condition LST. One may now speculate that odor cueing reduces the amount of forgetting over at least one week. However, this speculation holds only, if one also speculates that Condition N contains by accident more above average good learners than the other conditions. Whether this is the case and whether odor cueing really reduces forgetting over at least one week but not over four weeks, can only be answered more precisely in a follow-up repetition of our study.

### Possible reasons for only partial replication of previous TMR findings

Recent TMR studies with odor cueing reported positive memory effects with cueing during learning and during sleep (but not during testing, i.e. our LS Condition) compared to control conditions^23,30,40–42^. This finding initially defined the TMR effect. We did not find statistically significant differences between our LS Condition on one hand and the LT Condition (cueing during learning and during testing) and the N Condition (vehicle condition without odor). How can this replication failure be explained?

The present study differed in a number of parameters from the previous studies mentioned above. In a study design with separate groups for each of the experimental conditions (between-design) it is always possible that a significant number of good learners enter one group and a significant number of poor learners enter another group, independent of the experimental treatment (here the odor cueing). In the present study Condition N seem to contain a number of very good learners. Such potential confounds can theoretically be avoided in a within-design (all participants perform all experimental conditions). This would allow within-participant comparisons but comes with a number of other problems, as discussed in Neumann et al. Alternatively, one can try to reduce the problem by eliminating data points at the extreme ends of the distributions in a predefined reference condition to make the distributions comparable, as it was done in the present study.

In the following we speculate about two further potential candidates for an explanation. For each individual person we postulate an individually limited learning capacity, given a learning task with a certain amount of learning materials, a certain time window of a Learning Session and a fixed number of repetitive Sessions. This limited learning capacity may change across the successive Learning Sessions, as a function of how much has already been memorized and how much still needs to be memorized. We further postulate that the more this limit is reached, the less odor can unfold its impact.

In the present study our participants had to learn 40 German-Japanese vocabulary pairs. One important point may be that they saw the 40 word-pairs and were tested on them two times in succession in the initial two Learning Sessions on Day 1. This may have already evoked a relatively high learning level, potentially close to the participants’ individual capacity limits, preventing the odor cueing effect to unfold in full size. Such a pattern can be observed in the Fig. S4 (in the Supplementary Materials), where the memory performances of Intermediate Tests 1 and 2 (at the same day) and Intermediate Test 3 immediately after a night of sleep are compared.

Another important factor in our experimental design are the Intermediate Tests during the learning period. Aim of these tests was, to measure the learning dynamics in the learning period across the three days. The well-known “Testing Effect”^21,43–45^, as introduced above may play a considerable role in this context. The Intermediate Tests during our learning period most probably have introduced a Testing Effect on the learning success of our participants. Moreover, this postulated Testing Effect may have moved the individual learning dynamics close to the individual capacity limits (see the paragraph above concerning individual capacity limits), again or additionally preventing the odor cueing effect to unfold in full size. These considerations are in line with a recent study by Joensen et al.^29^, showing the influence of intermediate tests on TMR effects.

On this background it is even more remarkable that we found a TMR effect in the LST Condition. One possible reason for this may be that the mechanisms underlying the beneficial effect of odor cueing on consolidation are separate from those underlying beneficial effect on retrieval. The beneficial effect on retrieval may be unaffected by the individual learning capacity limits. The beneficial effect of odor cueing on consolidation, in contrast, may be absent because the Testing Effect may have already pushed the system close to its learning capacity limit, as explained above. It is thus possible that our findings in the LST Condition shows the isolated TMR effect on retrieval.

If our above speculation turns out to be correct, we should expect also a–yet smaller—effect in the LT Condition (odor cueing during learning and retrieval but not during sleep). Some studies indeed reported that the presentation of odor during learning and retrieval helps to recall memories^46,47^. In the present study the memory performance in the LT Condition should thus be between that of the N Condition and the LS Condition. However, we did not find any TMR effect in the LT Condition. A possible reason for this may be that our N Condition contained some good learners, and the LT Condition perhaps more poor learners, as already discussed above. Those exceptional learners may have not been detected with our data correction method on T2 data.

## Conclusion

The amount of information that enters our senses extends by far our memory capacities. We – or at least some instances within us – need to select relevant information, worth being memorized, from irrelevant information that can be forgotten. The criteria for this selection are only partly understood. The TMR effect is a fascinating tool to gain some more influence on this selection.

Most of the research about learning and memory and about the TMR effect had been executed in labs with highly controlled and isolated experimental conditions. Maximal control is necessary to rule out as much potential confound as possible. However, it is always a challenge to draw practical conclusions from such artificial situations. One merit of the current study and Neumann et al. is the demonstration that the olfactory TMR effect obviously works in real-life situations in a school environment and at the participants’ home. Particularly, the two studies demonstrate that odor cueing during the whole night works as well as selected odor stimulation during specific sleep periods. This finding is important because it proves effortful sleep-monitoring as unnecessary for the practical application (see Whitmore et al.^28^ for an approach with a temporally specified stimulation during sleep in an auditory TMR paradigm).

Beyond the present proof of principle, several questions remain. We still don’t know the optimal number of Learning Sessions, the optimal amount of learning content, the best working type and intensity of odor, the optimal time point of the Learning Session before sleep etc. It is also unclear whether TMR works across all ages and whether we can expect adaptation-like effects if we apply TMR too often.

The TMR topic is still in its infancy and much more research is necessary to better understand the necessary framing conditions and the underlying mechanisms. However, the present findings already indicate that TMR is an unexpensive and easy-to-apply tool that can be used by almost everyone.

## Supplementary Information

Supplementary Information.

## Data Availability

The datasets used and analyzed during the current study are available from the corresponding author on reasonable request.
