# Supplementary material for: Presenting rose odor during learning, sleep and retrieval helps to improve memory consolidation: a real-life study
Source: Sci Rep. 2023 Feb 9;13:2371. doi: 10.1038/s41598-023-28676-z (PMC9911722; doi:10.1038/s41598-023-28676-z)
Supplement: Supplementary file 1 — Supplementary Information. [file 41598_2023_28676_MOESM1_ESM.pdf]

Presenting rose odor during learning, sleep and retrieval helps to improve memory consolidation- a real-life study

Supplementary Materials

Jessica Knötzele[1,2,3], Dieter Riemann[2,3], Lukas Frase[2,3], Bernd Feige[2,3], Ludger Tebartz van Elst[2,3], Jürgen Kornmeier[1,2,3,*]

[1]Institute for Frontier Areas of Psychology and Mental Health, Freiburg, Germany;
[2]Department of Psychiatry and Psychotherapy, Medical Center - University of Freiburg, ;
[3]Faculty of Medicine, University of Freiburg, Germany

*Corresponding Author

Table S1. Vocabulary pairs

German word	Japanese word	German word	Japanese word
Braten (roast)	yakiniku	wohlhabend (prosperous)	yūfuku
Garderobe (wardrobe)	kurōku	Foto (photo)	shashin
Urlaub (holiday)	kyūka	Geschenk (gift)	okurimono
Bildung (education)	kyōyō	Brille (glasses)	megane
Küche (kitchen)	daidokoro	Reden (talk)	hanasu
Höhe (height)	takasa	kochen (cook)	tsukuru
Schere (scissors)	hasami	Marke (brand)	burando
Schlafen (sleep)	nemuru	lernen (learn)	manabu
erwidern (respond)	kotaeru	unbekannt (unknown)	shiranai
abweichen (deviate)	soreru	Angler (angler)	tsuribito
fühlen (feel)	kanjiru	beharrlich (persistent)	nebarizuyoi
zierlich (dainty)	kyasha	freundlich (kind)	yasashii
Eltern (parents)	ryōshin	fordern (demand)	motomeru
weiblich (female)	onnarashii	finden (find)	mitsukeru
Mahnung (reminder)	keikoku	Schrank (cabinet)	todana
Fehler (mistake)	machigai	Unheimlich (sinister)	monosugoku
färben (dye)	someru	deutlich (clear)	wakariyasui
Kerze (candle)	rōsoku	Beweglich (flexible)	ugokaseru
Paradies (paradise)	rakuen	Gehalt (salary)	kyūryō
Pflanze (plant)	shokubutsu	unregelmäßig (irregular)	fukisoku

Table S1: German-Japanese vocabulary pairs. Shown are the vocabularies, which were used in this study. The English translation is displayed in parentheses after the German Words.

Table S2. Basic statistics for Intermediate Test 2

	LST		N		LS		LT	
**Raw data**	N:	42	N:	41	N:	41	N:	41
	Mean:	9.1667	Mean:	10.488	Mean:	8.3659	Mean:	11.39
	SD:	5.3003	SD:	6.2094	SD:	4.7841	SD:	6.2765
	SEM:	0.81786	SEM:	0.96974	SEM:	0.74715	SEM:	0.98022
	Max:	26	Max:	30	Max:	18	Max:	24
	Min:	0	Min:	2	Min:	0	Min:	1
	Median:	8	Median:	9	Median:	8	Median:	11
	LQ:	6	LQ:	7	LQ:	4	LQ:	6
	UQ:	13	UQ:	14	UQ:	11	UQ:	16
**Corrected data**	N:	34	N:	37	N:	36	N:	39
	Mean:	11	Mean:	9.4595	Mean:	9.3333	Mean:	9.2051
	SD:	5.0151	SD:	4.3627	SD:	4.2628	SD:	4.2931
	SEM:	0.86009	SEM:	0.71722	SEM:	0.71047	SEM:	0.68744
	Max:	19	Max:	19	Max:	18	Max:	19
	Min:	3	Min:	3	Min:	3	Min:	3
	Median:	11	Median:	9	Median:	9	Median:	8
	LQ:	6	LQ:	7	LQ:	7	LQ:	6
	UQ:	15.25	UQ:	13	UQ:	12.25	UQ:	13

Table S2: Basic statistics. The results of basic statistics are shown for each condition (from left to right: LST, N, LS, LT) for Test 2. The upper part of the table displays the statistical results of the raw data, the lower part of the outlier-corrected data (minus 5% of the best and the worst performing participants). N represents the number of participants included in the respective condition. Moreover, the Mean (grand mean), the SD (standard deviation), SEM (standard error of the mean), the Max (maximum of correct words), the Min (minimum of correct words) and the Median are listed. LQ indicates the lower quartile, UQ indicates the upper quartile.

Table S3. Statistics for the sleep times (see Figure 3)

	N	LS	LT
**Night 1**			
**LST**	0.609	0.222	0.562
**N**		0.819	0.544
**LS**			0.831
**Night 2**			
**LST**	0.883	0.835	0.812
**N**		0.602	0.665
**LS**			0.461
**Night 3**			
**LST**	0.618	0.52	0.987
**N**		0.551	0.041
**LS**			**0.957**

Table S3: Uncorrected Post-hoc tests comparing sleep times of the first three nights.

Table S4. Statistics for the cumulative delays in start time of the Learning Period across days (Figure S2)

	N	LS	LT
**LST**	0.845	0.811	0.253
**N**		0.499	0.942
**LS**			0.102

Table S4: Statistics for the cumulative delays in start time of the Learning Period. Presented are the uncorrected p-values between all conditions separately. No systematic difference in delay can be detected.

Table S5 Statistics of the uncorrected data

	LT	LS	LST
**T1 on Day 1**			
**N**	0.15, (0.22)	0.86, (0.22)	0.23, (0.14)
**LT**		0.96, (0.39)	0.64, (0.081)
**LS**			0.076, (0.32)
**T2 on Day 1**			
**N**	0.84, (0.23)	0.96, (0.38)	0.28, (0.14)
**LT**		0.77, (0.16)	**0.043, (0.38)**
**LS**			**0.003, (0.54)**
**T3 on Day 2**			
**N**	0.93, (0.33)	0.93, (0.33)	0.18, (0.2060)
**LT**		0.47, (0.0023)	**0.01, (0.51)**
**LS**			**0.014, (0.50)**
**T4 on Day 3**			
**N**	0.94, (0.33)	0.91, (0.31)	0.19, (0.18)
**LT**		0.52, (0.0057)	**0.011, (0.51)**
**LS**			**0.018, (0.47)**
**F1 on Day 4**			
**N**	0.83, (0.22)	0.92, (0.32)	0.1, (0.27)
**LT**		0.27, (0.12)	**0.014, (0.49)**
**LS**			**0.003, (0.57)**
**F2 on Day 10**			
**N**	0.98, (0.52)	0.95, (0.35)	0.27, (0.12)
**LT**		0.27, (0.14)	**0.002, (0.61)**
**LS**			**0.021, (0.44)**
**F3 on Day 31**			
**N**	0.97, (0.40)	0.99, (0.60)	0.43, (0.034)
**LT**		0.82, (0.21)	**0.027, (0.41)**
**LS**			**0.002, (0.57)**

Table S5: Uncorrected post-hoc tests for intermediate tests and Final Tests based on the uncorrected data (concerning outliers). The results of the permutation tests are displayed for each vocabulary test separately (from top to bottom: T1, T2, T3, T4, F1, F2, F3). The statistically significant p-values and effect sizes (Cohen's D in parentheses) are displayed in bold.

Table S6 Statistics of the slope of the linear fits

**Comparing Slopes of linear fits from Test 2 (Day 1) to Final Test 1(Day 4)**			
	**N**	**LS**	**LT**
**LST**	0.056, (0.36)	0.07, (0.35)	**0.02, (0.51)**
**N**		0.48, (0.005)	0.29, (0.13)
**LS**			0.31, (0.12)
**Comparing Slopes of linear fits from Test 3 (Day 2) to Final Test 1(Day 4)**			
	**N**	**LS**	**LT**
**LST**	**0.024, (0.45)**	**0.024 (0.44)**	**0.007, (0.57)**
**N**		0.42, (0.034)	0.27, (0.13)
**LS**			0.36, (0.084)

Table S6: Permutation test statistics of the slope parameter between conditions. Listed are p-values and Cohen's d for significant p-values in parentheses. Significant p-values (alpha = 0.05, with Bonferroni-Holm correction) and effect sizes in bold.

Figure S1. Cumulative delays in start time of the Learning Period across days

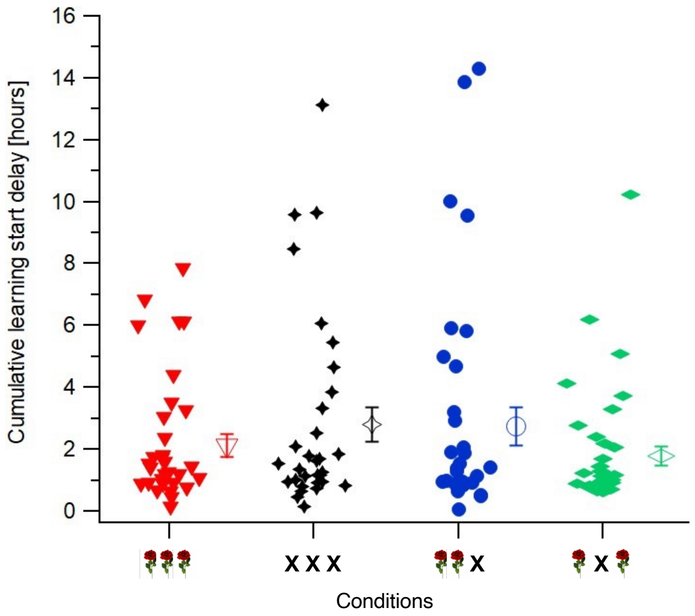

Figure S1: Cumulative delays in start time of the Learning Period across days. The starting time point (time of the day) of the first Learning Session at the first day defined for each participant individually her/his starting point for the subsequent two Learning Sessions on the subsequent two days. This means that if an example participant started at 8 am on the first day she/he was supposed to also start at 8 am on days 2 and 3. The cumulative delay in start time of this participant represents the sum of delays of the starting times on days 2 and 3 with respect to the starting time at day 1. The different conditions (LST in red, N in black, LS in blue and LT in green) are displayed on the x-axis. The time in hours is shown on the y-axis. Each filled icon represents one participant. The grand means (larger unfilled icons) ± SEMs are displayed on the right-hand side of each cloud of icons. Shown are all participants of the outlier-corrected data.

Figure S2. Comparison between Intermediate Tests 1, 2 and 3 from the Learning Period

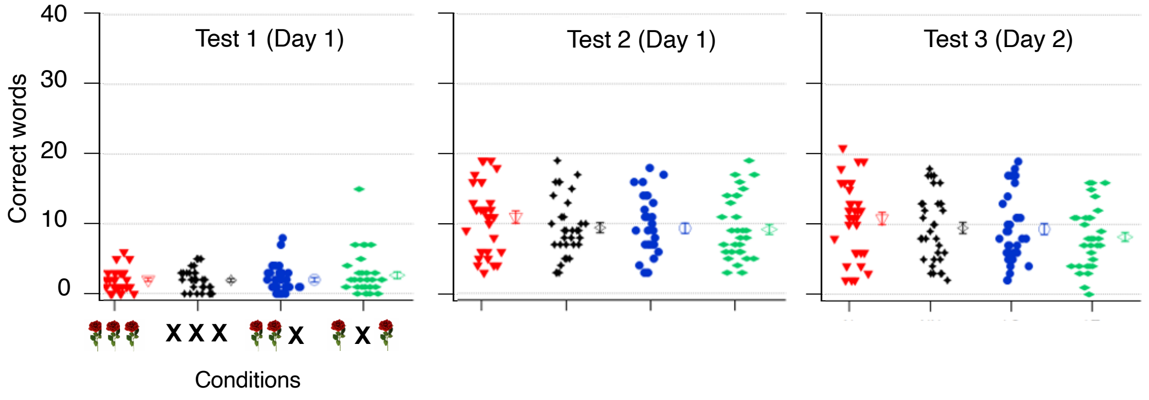

Figure S2: Corrected results from the Intermediate Tests 1 and 2 of Learning Session 1 and 2 on Day 1 and from Learning Session 3 on Day 2 after the first night of odor stimulation in conditions LS and LST. The different conditions (LST in red, N in black, LS in blue and LT in green) are displayed on the x-axis, the number of correctly remembered words on the y-axis. Each filled icon represents one participant. The grand means (larger unfilled icons) ± SEMs are displayed on the right-hand side of each cloud of icons. Notice that the major learning step takes place between intermediate Tests 1 and 2 but not over night between Test 2 and 3. We explain this with a potential limitation of the learning capacity (i.e. a kind of ceiling effect), as explained in the Discussion section.

Figure S3. Results from Intermediate Tests during the Learning Period.

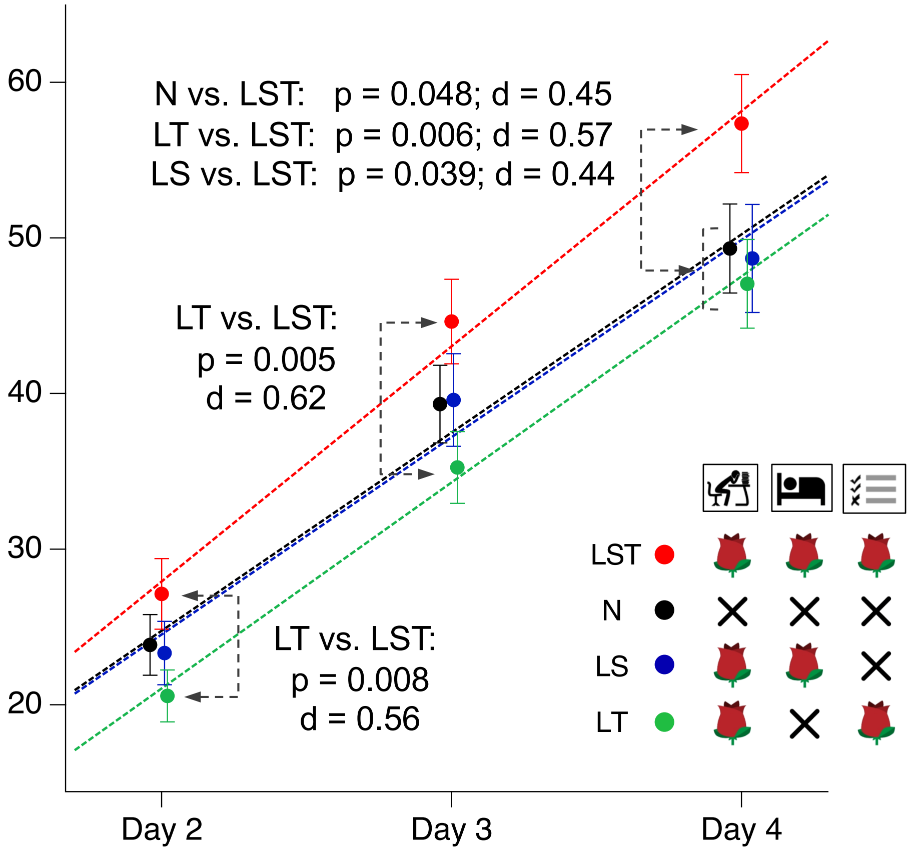

Figure S3. Results from the Intermediate Tests during the Learning Period. Grand means per condition and day with linear fits and results from the post-hoc tests (same as in Fig. 4). Please notice that the presented fits are only for demonstration purposes. For the data analysis we used the slope parameter from individual linear fits separately for the individual participants. We restricted the linear fits on data from Intermediate Tests 3 (on day 2) to Final Test 1 on day 4 and ignored the Intermediate Tests from day 1 (see the related discussion in the paragraph “Limitations of this study”). d: Cohen’s d.

Figure S4 Comparison between raw and corrected data

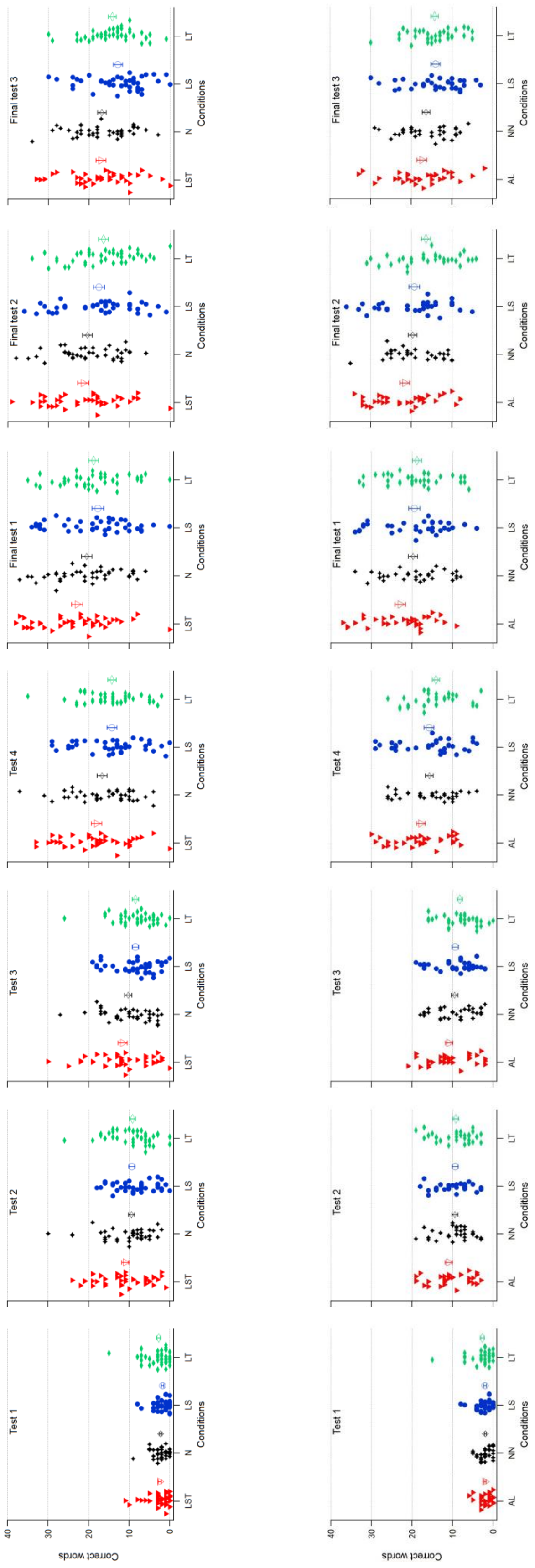

Figure S4: Number of correct remembered words for the different experimental conditions. Top row: uncorrected data, bottom row: data corrected for outliers. The different graphs in a row represent the four Intermediate Tests from the Learning Period and the three Final Tests from the Final Test Period. The different conditions (LST in red, N in black, LS in blue and LT in dashed green) are displayed on the x-axis. The number of correct words is displayed on the y-axis. Each filled icon represents one participant. The grand means (larger unfilled icons) ± SEMs are displayed on the right-hand side of each cloud of icons.

Acknowledgement

Very special thanks go to the Noaks Company, who kindly donated us a large quantity of fragrance-impermeable Noak bags. We would also like to thank the Kohnert GmbH for providing us with the odor sachets at purchase price. Thanks also to the Gorilla Platform team for believing in our project. By winning the second place of the Gorilla Grant, the participation of all test persons on the platform could be financed. Furthermore, I would like to thank all test persons who made the realization of this online study possible by their participation!
